# More frequent naps are associated with lower cognitive development in a cohort of 8–38‐month‐old children, during the Covid‐19 pandemic

**DOI:** 10.1002/jcv2.12190

**Published:** 2023-07-27

**Authors:** Teodora Gliga, Alexandra Hendry, Shannon P. Kong, Ben Ewing, Catherine Davies, Michelle McGillion, Nayeli Gonzalez‐Gomez

**Affiliations:** ^1^ School of Psychology University of East Anglia Norwich UK; ^2^ Department of Experimental Psychology University of Oxford Oxford UK; ^3^ Centre for Psychological Research Oxford Brookes University Oxford UK; ^4^ School of Languages, Cultures and Societies University of Leeds Leeds UK; ^5^ Department of Psychology University of Warwick Coventry UK

**Keywords:** Covid‐19, executive functions, napping, pre‐school children, sleep, vocabulary

## Abstract

**Background:**

How often a child naps, during infancy, is believed to reflect both intrinsic factors, that is, the need of an immature brain to consolidate information soon after it is acquired, and environmental factors. Difficulty accounting for important environmental factors that interfere with a child's sleep needs (e.g., attending daycare) has clouded our ability to understand the role of intrinsic drivers of napping frequency.

**Methods:**

Here we investigate sleep patterns in association with two measures of cognitive ability, vocabulary size, measured with the Oxford‐Communicative Development Inventory (*N* = 298) and cognitive executive functions (EF), measured with the Early EF Questionnaire (*N* = 463), in a cohort of 8–38‐month‐olds. Importantly, because of the social distancing measures imposed during the Covid‐19 Spring 2020 lockdown, in the UK, measures of sleep were taken when children did not access daycare settings.

**Results:**

We find that children with more frequent but shorter naps than expected for their age had lower concurrent receptive vocabularies, lower cognitive EF and a slower increase in expressive vocabulary from spring to winter 2020, when age, sex, and SES were accounted for. The negative association between vocabulary and frequency of naps became stronger with age.

**Conclusions:**

These findings suggest that the structure of daytime sleep is an indicator of cognitive development and highlight the importance of considering environmental perturbations and age when investigating developmental correlates of sleep.

## Graphical abstract

Frequent napping, in childhood, is believed to reflect an brain's need to consolidate information soon after learning, yet a previous study showed that more frequent napping associates with higher rather than lower cognitive development. During the UK Covid‐19 lockdown, closure of daycare removed one environmental factor that may interfere with children's sleep need. In a cohort of 8–38‐month olds followed up during lockdown, more frequent naps associated with lower concurrent vocabulary and executive functions. For example, still napping once a day, in children over 25 months old, is associated with lower vocabularies (see Figure).

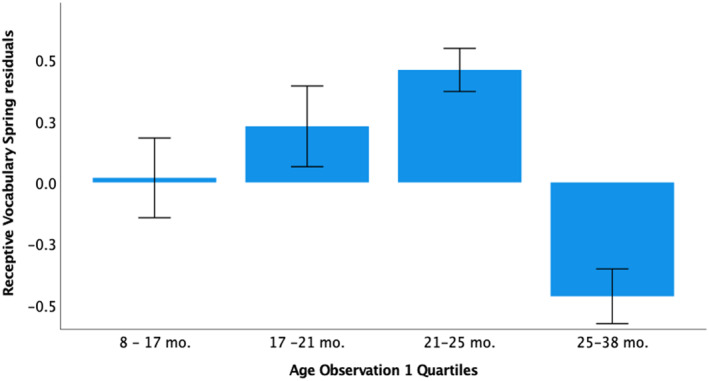

**Key points**

- Frequent napping, in infancy, is believed to reflect an immature need to consolidate information soon after learning, yet a previous study showed that more frequent napping associates with higher rather than lower cognitive development
- During the UK Covid‐19 lockdown, closure of childcare removed one environmental factor that may interfere with children's sleep need
- We show that, in a cohort of 8–38‐month olds followed up during lockdown, more frequent naps associated with lower concurrent vocabulary and executive functions (EF).
- A child's cognitive development level influences how often they needs to nap
- Early years practice should use a child's mental and not chronological age to determine a child's sleep needs.

## INTRODUCTION

Sleep changes dramatically during development, with early infancy seeing a universal pattern of gradual establishment of day and night sleep rhythm and subsequent development characterized by consolidation of daytime sleep into a decreasing number of naps of longer duration (Blair, Humphreys & Gringas, 2012; Galland et al., 2012; Staton et al., 2020). While these transitions may reflect the consolidation of circadian rhythms, and their complex neural‐hormonal contributions (Logan & McClung, 2019; Rivkees, 2003), more recently the view has emerged that frequent napping, during infancy, may satisfy the needs of an immature memory consolidation system (e.g., Gómez & Edgin, 2016; Kurdziel et al., 2013). Indeed, complementing a rich literature that supports the role of sleep for information consolidation in adults (for a review see Rasch & Born, 2013), there is accumulating evidence that naps benefit learning in infancy and that consolidation of information depends on sleep occurring soon after learning. For example, 6‐ and 12‐month‐old infants retained a sequence of imitative actions when napping within a 4‐h interval after learning, with no extra gain from a subsequent night's sleep (Seehagen et al., 2015). Fifteen‐month‐olds learned and generalized new grammatical patterns when a nap followed within 4 h of learning (Hupbach et al., 2009). Newly walking infants, become more efficient in a new motor task after a nap, compared to a no nap group (Berger & Scher, 2017; Horger et al., 2023). Again, the timing of the nap mattered for learning this motor task, with benefits seen only when napping within a 4‐h window (DeMasi et al., 2021). In contrast to these studies, napping in 3–5‐year‐old children, who do not naturally nap, was shown to have little impact on information consolidation (Kurdziel et al., 2013).

While the time course of changes in nap structure is universal, with few cross‐cultural differences (Mindell et al., 2011; Staton et al., 2020), there is a large individual variation in how long and how frequently children sleep during the day, at any point in development. For example, most 2‐year‐olds nap once daily, but some still nap twice a day and others only nap occasionally. Given the above suggestion that changes in the frequency of naps may index the maturation of information consolidation networks, napping more frequently than expected for a particular age may be an indication of developmental delay (we will refer to this as the *maturational* model of sleep). Thus, a negative association is expected between the age‐adjusted frequency of naps and development level. In line with this prediction, Dionne et al. (2011) found that less consolidated sleep at 6 and 18 months (a higher proportion of day to night sleep) was associated with language delays at 60 months; this study did not explicitly investigate the impact of the number of naps, however. In contrast, Horváth and Plunkett (2016) found that more frequent naps, in a cohort of 246 8–36‐months‐olds, predicted larger concurrent vocabularies and larger vocabulary growth over a period of 3 months. These authors interpreted their findings in terms of a *sleep deficit* model, where variation in the number of naps is a result of extraneous factors that prevent children from fulfilling their sleep needs, with negative consequences for learning. Indeed, whether a negative or positive association is revealed between nap frequency and developmental measures depends very much on whether children nap as frequently as they need to. Various factors may interfere with this need being fulfilled, one such factor being whether the child attends a daycare setting. Accommodating individual children's sleep needs is challenging in daycare settings that often mix children with different nap needs (Thorpe et al., 2020; Ward et al., 2008) and decreases in daytime sleep are reported when children transition to daycare (e.g. Cairns & Harsh, 2014; but also see Newton & Reid, 2023 for no association between childcare attendance and children's nap profiles). Parents themselves sometimes express a preference for their children not to nap when in childcare, in order for them to have earlier bedtimes (Sinclair et al., 2016). In the UK 3–5‐year‐olds are often enrolled into preschools, where there is typically no provision for napping, even though about half of 3‐year‐olds would nap if given the opportunity. Although no formal analysis of the influence that childcare attendance has on napping patterns was carried out by Horváth & Plunkett, 2016, about 50 percent of this cohort attended childcare (Horvath, personal communication), which may, in part, explain their findings.

To test whether the number of naps may act as an index of a cognitive need for sleep, we should, ideally, measure napping in children that do not attend childcare. Recruiting a large enough sample of children with no childcare attendance is problematic because the sample would not be representative, with socioeconomic status being a strong associate of childcare attendance (Department for Education [DfE], 2019; Davies et al., 2021). The closure of daycares and the social distancing measures imposed during the Covid‐19 lockdown in the UK, in the spring 2020, offered a unique opportunity to investigate the association between the number of naps and cognition in a large sample of 8–38‐month‐old children, a similar age range as that in Horváth & Plunkett, 2016. As in this previous paper, the current study investigated the growth in receptive and expressive vocabulary, measured with the Oxford‐CDI (Hamilton et al., 2000). In addition, to test for the generalizability of our hypothesis, we also investigated the association between sleep and cognitive EF. Cognitive aspects of EF encompass inhibitory control, flexibility, and working memory. While memory consolidation has repeatedly been linked to the need for sleep, the link with EF may seem less straightforward. However, a case was made recently that the development of executive processes relies on acquiring (and therefore potentially consolidating during sleep) knowledge, beliefs, norms, and values (Doebel, 2020). A growing number of studies has linked variation in day sleep and the growth in EF (Breitenstein et al., 2021). In line with a positive association between sleep and cognition (the sleep deficit model), Bernier et al. (2013) found that shorter day sleep at 1 year of age is associated with lower EF at 4 years of age. In contrast, Morales‐Munoz et al. (2021) described an inverted U‐shaped association between the proportion of day to night sleep at 1 year of age and inhibitory control 2 years later, which can only be explained by appealing to both the *sleep deficit* and the *maturational* models of sleep. In support of the maturational view, Lam et al. (2011) described a negative association between napping frequency and a number recall test, which requires attention and working memory. To quantify executive control, we used the Early EF Questionnaire, a recently developed parent report questionnaire (Hendry & Holmboe, 2021) which captures growth in these skills in pre‐school children.

**FIGURE 1:**
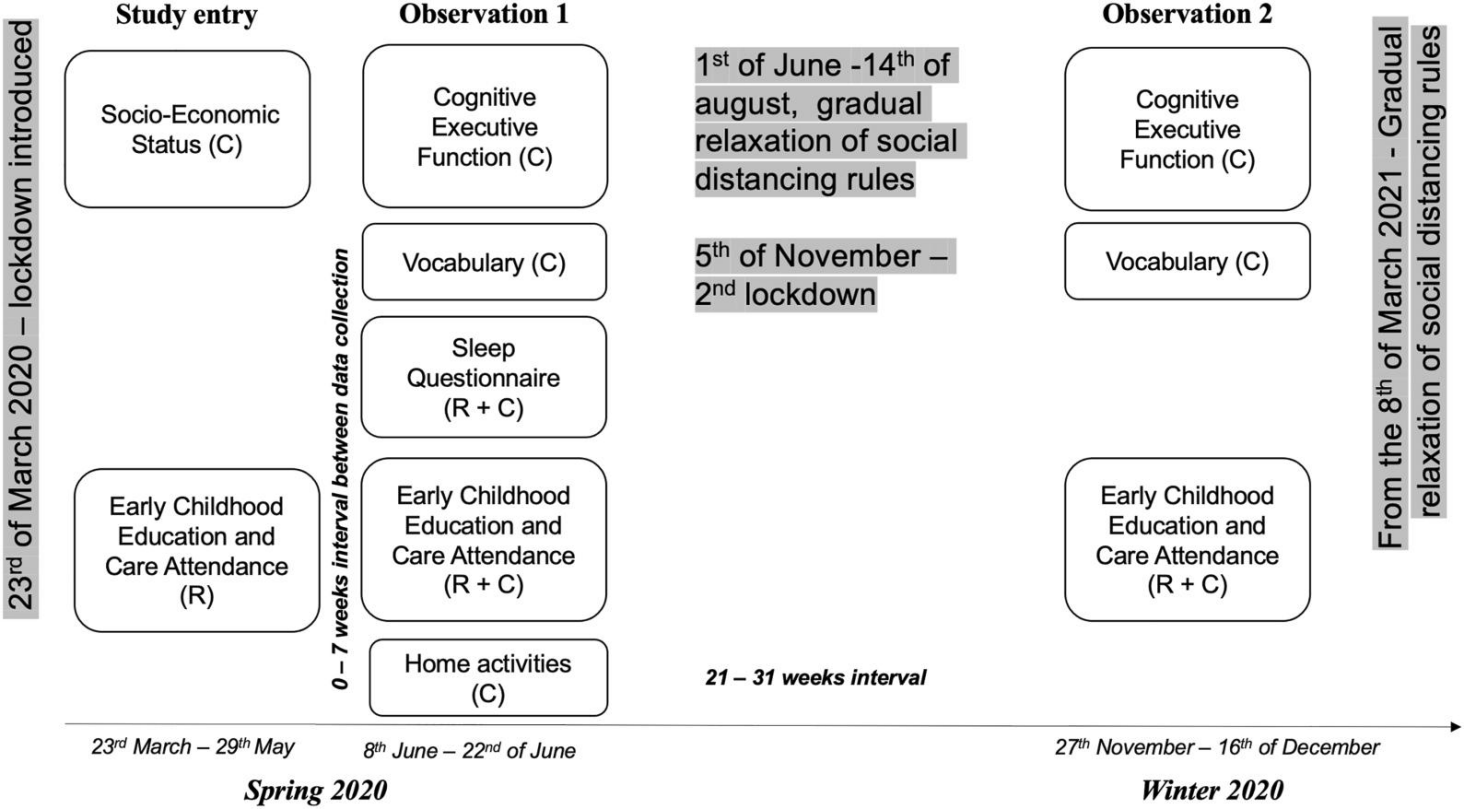
Overview of study measures by time point, indicating which measures were collected retrospectively (R) and which concurrently (C). Data collection intervals are indicated as well as the time lag between data collection waves. Key UK dates for the onset or relaxation of social distancing measures are indicated for reference.

Participants in the current study were recruited in spring 2020, as the UK entered a period of social distancing restrictions, which also included the closure of daycare centers for most children (Figure 1). Measures of sleep and cognitive development were collected at a first time point, 11–13 weeks after the onset of lockdown (Observation 1), and we focus here on the subset of children that had no access to childcare during this interval. The cohort was followed up in winter 2020, when vocabulary and executive function measures were again collected (Observation 2). This dataset allows us to test for both concurrent associations between sleep variables and cognitive development collected at Observation 1, as well as for longitudinal associations with the change in vocabulary and EF score between spring and winter of the same year. To account for individual variation in nighttime sleep, which may also be associated with cognition, we include in analysis key measures of day (the number and length of naps) and night sleep (length and efficiency, Mason et al., 2021). Based on the assumption that during spring lockdown, when children did not attend childcare, they were allowed to nap as frequently as they needed (partially supported by our own data, see below) we expect to describe a negative concurrent association between the frequency of naps, standardised for age, and vocabulary/EF. Assuming little individual variation in the rate of maturation, the maturational view of sleep would predict no association between the number of naps and the growth in vocabulary and EF between the two observations. Because we have already reported that in this cohort, decreased access to childcare during lockdown had a negative impact on vocabulary growth in those children coming from lower socioeconomic status (SES) families (Davies et al., 2021; Hendry et al., 2022), SES was used as covariate in all analyses, to account for environmental factors that may affect both sleep and cognitive development. The amount of screen time and the amount of time spent outdoors were previously associated with variation in sleep patterns, throughout lifespan (Cheung et al., 2017; Pilz et al., 2018). Given reports of children spending more time with screens and less outdoors during Covid‐19 lockdown (Bergmann et al., 2022; Kartushina et al., 2022), we also explored the association between these two variables and sleep.

## MATERIALS and METHODS

### Participants

Families with infants and children under 36 months of age were recruited from across the UK to take part in a study on cognitive development during the 2020 Covid‐19 lockdowns. Participants were recruited through online advertisements on research‐related websites and social media groups. Eight hundred and sixty‐two participants were recruited between 23 March and 29 May 2020 (*Spring 2020*; 0–67 days after the initiation of UK lockdown measures). An additional 1 child had a genetic condition, 4 were not living in the UK, 23 were born at less than 37 weeks gestational age, and 2 infants were siblings whose data were incorrectly entered at a later timepoint; these infants were excluded from analyses. Ninety‐nine per cent of respondents were the target child's mother, 1% their father.

A subset of these children was included in the analyses presented in this paper. Part of the attrition is due to parents not filling one or more of the questionnaires included in the current analysis. Only monolingual infants were included in the analysis linking sleep and language development. A small proportion of children were excluded because they had access to childcare during spring 2020 lockdown. Two children were excluded because of being outliers on Age at Observation 1, that is, 42 and 45 month old. These participants did not contribute vocabulary data. Demographics for children included in various analyses are presented in Tables 1 and 2.

**TABLE 1:** Descriptive statistics (means, min and max values) for the predictors used in the Concurrent analysis (measures collected at Observation 1).

Mean (min, max)	Vocabulary	Executive functions
Age (days)	608.38 (241, 1159)	619.91 (241, 1159)
Age (months)	20 (7.9, 38)	20.3 (7.9, 38)
SES	0.04 (−2.55, 2.00)	0.03 (−3.02, 2.00)
Sex (female/male)	145/153	230/231
Number of naps	1.14 (0, 2)	1.12 (0, 3)
Nap duration (hours)	1.30 (0, 2.00)	1.27 (0.00, 2.00)
Sleep efficiency	0.96 (0.55, 1.00)	0.96 (0.55, 1.00)
Time asleep night (hours)	10.86 (4.25, 13.25)	10.83 (4.25, 13.50)
Receptive vocabulary (count)	264.63 (3, 416)	‐
Expressive vocabulary (count)	161.05 (0, 416)	‐
Cognitive executive functions (CEF)	‐	4.65 (2.27, 6.60)
*N*	298	461

*Note*: Because different samples were entered in the analysis for Vocabulary and Executive Functions, these are described separately.

**TABLE 2:** Descriptive statistics for the predictors used in the Longitudinal analysis.

Mean (min, max)	Vocabulary	Executive functions
Age Obs 2 (days)^a^	824.60 (451, 1359)	820.21 (451, 1359)
Age Obs 2 (months)^a^	27 (14.7, 44.5)	26.9 (14.7, 44.5)
SES	0.04 (−2.55, 2.00)	0.05 (−2.55, 2.00)
Sex (female/male)	77/88	85/99
Number of naps Obs 1	1.12 (0, 2)	1.11 (0, 2)
Nap duration Obs 1	1.31 (0.00, 2.00)	1.29 (0.00, 2.00)
Sleep efficiency Obs 1	0.96 (0.77, 1.00)	0.96 (0.77, 1.00)
Time asleep night Obs 1	10.75 (5.16, 13.00)	10.64 (5.16, 13.00)
Receptive vocabulary Obs 2^a^	349.34 (53, 416)	‐
Expressive vocabulary Obs 2^a^	273.37 (0, 416)	‐
CEF Obs 2^a^	‐	4.98 (2.71, 6.27)
Receptive vocabulary change rate^b^	0.39 (0, 1.32)	‐
Expressive vocabulary change rate^b^	0.55 (0, 1.87)	‐
CEF change rate^b^	‐	0.0015 (−0.01, 01)
*N*	165	184

*Note*: Because different samples were entered in the analysis for Vocabulary and Executive Functions, these are described separately.

^a^ Not entered in the model but provided for reference.

^b^ Units per day (e.g. words acquired per day, CEF score points acquired per day).

This study received ethics approval from the Oxford Brookes University Research Ethics Committee. All procedures performed in this manuscript were in accordance with the 1964 Helsinki Declaration and its later amendments or comparable ethical standards. Participating caregivers provided informed consent at each timepoint, on behalf of themselves and their child. On completion Observation 1 measures, families were given a £30 Amazon voucher. On completion of the Observation 2 measures, families were given a £5 Amazon voucher.

### Measures

#### Socio‐economic status

An SES factor score was used, which had been calculated by Hendry et al. (2022), on the whole sample recruited for this study. Principal Components Analysis was used to extract this score from 4 indices, parental occupation score (0.828 factor loading), household income (0.823 factor loading), parental education score (0.781) and neighborhood deprivation index (0.528). Please see Appendix S2 for a detailed description of the 4 indices and Hendry et al. (2022) for a detailed description of the Principal Components Analysis analysis.

#### Early Childhood Education and Care attendance

Parents were asked whether their child received non‐parental childcare from a nursery, childcare setting, or nanny—henceforth ECEC—before (collected at Study entry) and during the spring lockdown (collected at Observation 1) and between Observation 1 and 2 (Pandemic Early Childhood Education and Care (ECEC)), and if so, to report the duration (full or half days), frequency (days per week), and degree of disruption (weeks prevented from accessing ECEC due to, for example, staff shortages, quarantining of close contacts); see Davies et al. (2021) for a full description of the measure. From this information, we computed the total number of days the child accessed ECEC before lockdown and during lockdown. Early Childhood Education and Care data were available for all except 1 participant, who indicated in the free text that they used a nursery but did not provide quantitative data and therefore were excluded from analyses. Only participants that did not attend childcare since lockdown onset and until data was collected at Observation 1, were included in analyses. Before lockdown, these children had attended on average 3 days/week (range 1–5). There was a gradual return to childcare from July and until the 2nd Observation was collected (see Figure S1 for a distribution of days spent in childcare between Observation 1 and 2).

#### Executive functions measure (EFs)

Parent‐report of emergent EFs was collected using the Early EF Questionnaire (EEFQ; Hendry & Holmboe, 2021). Data was collected concurrently at Observation 1 and 2. This questionnaire was developed and validated for children 8–30 months of age. Parents were asked to report how often in the last 2 weeks their child displayed particular EF‐related skills or behaviors using 28 questions with a 7‐item Likert response scale ranging from Never to Always: for example, “follow a simple instruction for a task that they were interested in (e.g., getting a nearby toy), without getting distracted.” Additionally, 3 games were included to elicit, in a semi‐standardised way, important EF‐related skills that might be difficult for parents to observe during casual play (such as holding in mind the location of a hidden item), or which might be context‐dependent (such as the child's ability to withhold a response when requested). Parents were asked to complete these games with their children and then score them (e.g. asking the child to wait before eating the food provided and timing for how long the child could wait, up to 30 s; for details see Hendry & Holmboe, 2021). In line with Hendry and Holmboe (2021) and Hendry et al. (2022), we computed a CEF composite score (20 questions, 3 games). Cognitive Executive Function scores were computed only where a minimum of 70% of applicable items were complete. Internal consistency was excellent for the CEF composite scales at both Observation 1 (Cronbach's alpha = 0.849) and Observation 2 (Cronbach's alpha = 0.835).

#### The Oxford‐CDI

The Oxford CDI (O‐CDI; Hamilton et al., 2000), is a caregiver‐report measure of child *receptive and expressive vocabulary*. The measure is commonly used with children up to 36 months of age. The O‐CDI was collected concurrently at Observation 1 and 2. Caregivers were asked to record whether their child “understood” or “understood and said” words from a list of 564 early vocabulary items. Receptive vocabulary was obtained by adding the “understood” and “understood and said” items.

#### Sleep measures

The sleep questionnaire was collected at Observation 1 and contained both concurrent and retrospective questions. We combined questions from the Brief Infant Sleep Questionnaire (Sadeh, 2004) and the Infant Sleep and Settle Questionnaire (Matthey, 2001). Parents were asked to report average estimates for the preceding 7 days for nap frequency, average nap duration, bedtime and wake‐up time for the night sleep, number of night awakenings and their average duration. In addition, we asked whether parents believed their child's sleep was affected by the social distancing measures, with specific options to choose between “No change”, “Sleeping less” or “Sleeping more”, asked separately for daytime and nighttime sleep. This data is retrospective and referred to as qualitative reports of lockdown impact on sleep duration.

Four quantitative measures of sleep were used in the analysis. The *number of naps*, took values between 0 and 3 (one child was reported to have 3 naps). The *time asleep at night* (in hours, with decimals) was computed as the difference in time between when the child woke up and when the child went to sleep at night, from which we subtracted the amount of time the child was awake at night. This latter measure was calculated by multiplying the number of night awakenings and the duration of each awakening. As in Horváth & Plunkett, 2016, night *sleep efficiency* was calculated as the ratio between the duration of night sleep and the total time spent in bed (the time between going to bed and waking up in the morning). *Nap duration* was the estimated duration of individual naps, expressed in hours. To account for large developmental variation in sleep patterns in our sample (see Figure S3), all 4 measures were standardised against age at Observation 1.

#### Home activities

Data was collected concurrently at Observation 1. Respondents were asked to report on the kinds of activities that they did with their child on a scale of 0 (‘Did not do at all’) to 9 (‘Performed this activity more than 4 h most days’). Questions were based on a measures developed to investigate the effects of COVID‐19 lockdowns in different countries (Bergmann et al., 2022; Kartushina et al., 2022). We calculated an Outdoors Activities score by summing the score for 4 items: time spent playing outdoors and time spent gardening, with or without the parent present. We calculated a Screen Use score by summing the score for each of the 6 activity items that involved watching TV or playing on a touchscreen. See Hendry et al. (2022) for more details on these questionnaires.

### Procedures and timeline

Questionnaires were administered online, via Qualtrics software. As shown in Figure 1, upon study entry, respondents answered questions relating to their socio‐economic characteristics (e.g., income, occupation, education), use of ECEC prior to lockdown, as well as several other aspects relevant only to the wider study. Data collection for Observation 1 took place zero to 7 weeks after completion of the study entry questionnaires, when participants had been in lockdown for at least 11 weeks. Participants reported on their child's EF skills, vocabulary (O‐CDI), postcode (as an additional SES indicator), ECEC, and filled in the Sleep Questionnaire, home activities as well as several other measures not relevant for this study. Twenty‐one to 31 weeks later, in Winter 2020, Observation 2 data was collected. Participants were asked to report again on their child's EF skills, vocabulary, ECEC and several other factors not investigated here; see Tables 1 and 2 for sample sizes of participants contributing data for the concurrent and longitudinal analyses.

### Preliminary analysis of confounding factors

Including SES as a covariate already accounts for various environmental factors that may perturb both sleep and cognitive development. However, several specific variables that were reported to change during lockdown, were known to impact both sleep and cognitive development. For example, a complex association has been found between more tablet use and more daytime sleep in infants, while more TV exposure has been associated with less daytime sleep (Cheung et al., 2017). Moreover, exposure to natural light modulates circadian rhythms (Pilz et al., 2018). Decreases in time spent outdoors were reported during the pandemic, including in our cohort (Hendry et al., 2022) and were associated with changes in sleep patterns (Korman et al., 2022), but a pre‐pandemic study found no association between time playing outdoors in childcare and children's sleep (Parsons et al., 2018). There are various reports of screen time increasing during lockdown (Aguilar‐Farias et al., 2021; Bergmann et al., 2022) and we previously reported that higher screen time was associated with lower CEF, in this cohort (Hendry et al., 2022). In addition, less time spent doing enriching activities, which included outdoors activities such as playing and gardening, was also associated with lower CEF (Hendry et al., 2022). However, in the current study, neither screen time nor time spent outdoors associated with various sleep measures, when age, gender, and SES were considered (see Table S1 for detailed analyses). It is possible that our measures do not capture well the main ingredients associated with sleep. When, during the day, children were outdoors or exposed to tables (whether just before nap or bedtime) may be more important than the total amount of time spent on these activities. These variables were therefore not included in further analyses.

Analytical approach. Three univariate ANOVAs were run to predict separately concurrent Cognitive EF, and Receptive and Expressive Vocabulary at Observation 1. Four sleep variables were entered as covariates: time asleep at night, sleep efficiency, individual nap length, and number of naps (all standardised against age). Age at Observation 1, sex, and SES were entered as additional covariates. To predict change in Cognitive EF and Receptive and Expressive Vocabulary, between Observation 1 and 2, rates of change scores were calculated for all these variables by dividing the difference between the measures taken at Observation 2 and 1 by the time delay between the two‐time points. Three univariate ANOVAs were run with rates of change as dependent variables and the same sleep variables at Observation 1 as above, age at Observation 1, Pandemic ECEC, SES, sex and the Observation 1 value for the key variable of interest (i.e., Cognitive EF at Observation 1 was entered as a covariate in the regression predicting change in Cognitive EF). Multicollinearity tests showed this assumption was met for all regressions. In a second step and to test for the moderating role of age in the association between sleep parameters and cognitive development, interaction terms between age and each of the sleep measure were added to each model. Significant interactions with age were illustrated in Supporting Information (see Figure S4) by scatterplots split by median age of 598 days, (approximately 20 months) which corresponds roughly to a transition between infancy (i.e. <20 month old) and toddlerhood (i.e. ≥20 month old).

## RESULTS

### Qualitative reports of lockdown impact on sleep

541 participants answered these questions which were part of the Sleep questionnaire at Observation 1, with 73.0% reporting no change in nap duration from before lockdown, 13.7% a decrease and 13.3% an increase in day sleep duration; 83.7% reported no change in the duration of night sleep while 9.6% reported shorter night sleep and 6.75% longer night sleep. Two multinomial logistic regressions were run, one for day sleep and one for night sleep to ascertain whether the amount of access to ECEC before lockdown, as well as the child's age or family SES impacted the likelihood of parents reporting an increase, a decrease or no change in sleep with lockdown. Before lockdown, children attended childcare in average 2.92 days (min 1 and max 5 days). Age had a significant impact on day sleep judgments (chi sq (2) = 7.61, *p* = 0.022) and so did SES (chi sq (2) = 8.16, *p* = 0.017) and pre‐pandemic ECEC (chi sq (2) = 8.08, *p* = 0.018). Those with a reported increase in day sleep were significantly younger than those with reported no change (*p* = 0.010) or with a decrease in day sleep (*p* = 0.025) and had been in ECEC for more days before lockdown than those with reported no change in (*p* = 0.017), with no difference when compared with those with a decrease in day sleep. The group with reported increase in day sleep also tended to come from higher SES families than those with a reported decrease in daytime sleep length (*p* = 0.060), with no difference when compared with those with no change in day sleep. Thus, a higher drop in the number of days attending ECEC was associated with both increases and decreases in the duration of day sleep, suggestive of our proposal that attending ECEC interferes with natural daytime sleep patterns. Only age associated with reported changes in nigh time sleep length (chi sq (2) = 10.18, *p* = 0.006). Those with reported increases in night sleep were significantly younger than those with reported no change (*p* = 0.004), or shorter night sleep (*p* = 0.007). This may reflect developmental change, with younger infants consolidating their sleep over the duration of the study.

Parental qualitative reports mapped onto differences in the reported length of day and night sleep. Children with a reported decrease in day sleep duration had fewer but longer naps during lockdown and children with a reported decrease in night sleep, slept for fewer hours and had lower sleep efficiency (see Appendix S1 & Table S2 for a detailed analysis).

### Concurrent associations between sleep and cognitive measures

#### Receptive and Expressive Vocabulary (Tables 1 & 3; Figure 2)

As seen in Table 3 (and in Figure S2), Age was strongly positively associated with both Receptive and Expressive Vocabulary while SES had a weak positive association only with Expressive Vocabulary. Less frequent naps and longer naps than expected for their age were associated with larger Receptive Vocabularies. Shorter time asleep at night was associated with larger Receptive Vocabularies. There were no significant associations between sleep variables and Expressive Vocabulary. The association between sleep parameters and vocabulary was moderated by age. The negative association with nap frequency and the positive association with nap length grew stronger with age, for both Receptive and Expressive Vocabularies (Figure S4).

**TABLE 3:** Concurrent effects at Observation 1.

Observation 1 measure	Receptive vocabulary	Expressive vocabulary	CEF
Model adjusted *R* ^2^	0.722**	0.702**	0.398**
Age	676.45**	649.08**	6.54**
SES	0.42	4.73*	6.08*
Sex	3.02	0.00	6.54*
Time asleep night	6.13*	0.11	0.32
Sleep efficiency	2.06	0.25	5.53*
Nap duration	33.66**	0.05	12.23**
Number of naps	24.74**	0.63	9.30**
Time asleep night × Age	0.15	2.70	1.69
Sleep efficiency × Age	0.06	4.50*	2.34
Nap duration × Age	8.41**	17.30**	0.02
Number of naps × Age	11.85**	30.63**	0.11
*N*	298	298	461

The table lists *F* values and significance level; **p < 0.01, *p ≤ 0.05. Model adjusted *R* ^2^ is reported for the main effects model.

**FIGURE 2:**
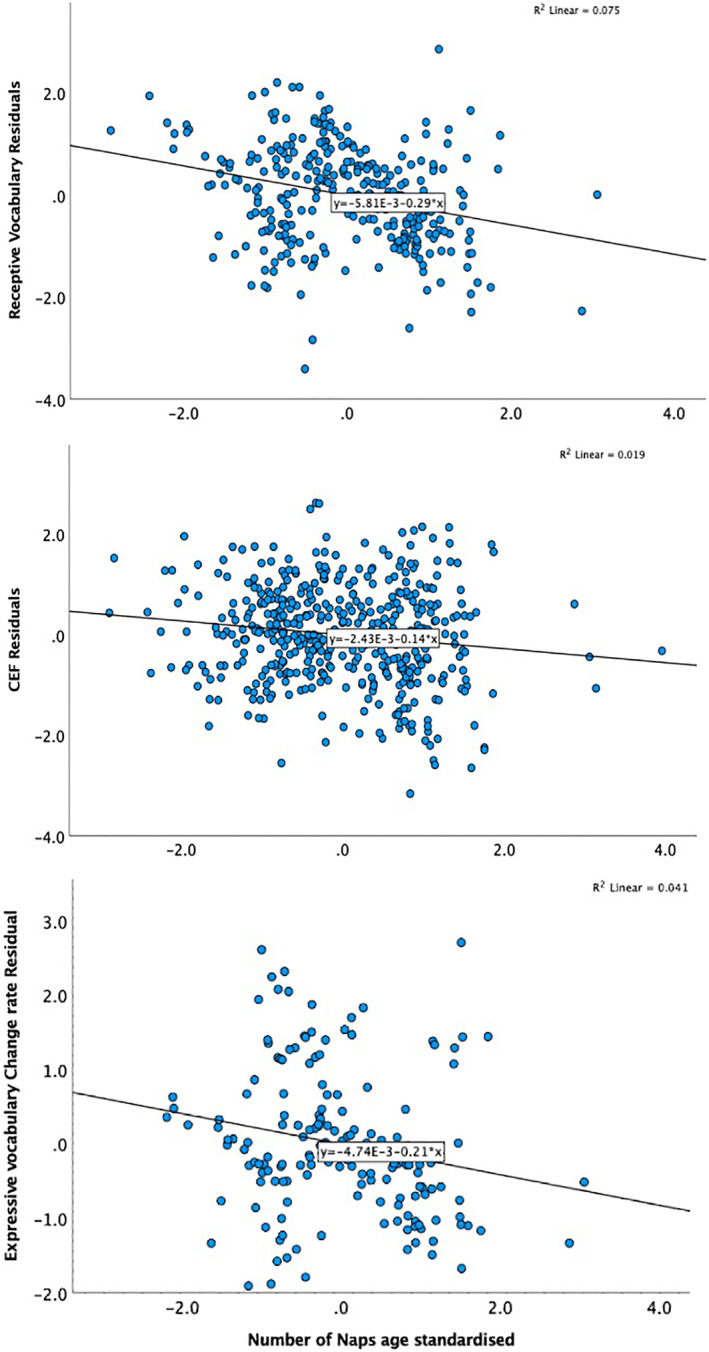
More frequent naps than expected for infants' age associates with lower Receptive vocabulary (top), Cognitive executive functions (EF) (middle) and with Expressive Vocabulary change rate (bottom). *Y*‐axes depict standardised residuals from the regression models reported in text, excluding the nap variable.

#### Cognitive executive functions (Tables 1 & 3, Figure 2)

Age was, as expected, a strong predictor of CEF (see Figure S2). SES was weakly and positively associated with CEF and girls tended to have higher CEF scores. Children that had shorter but more frequent naps than expected for their age, had lower CEF scores. More efficient night sleep was also positively associated with CEF. Age did not moderate the association between sleep parameters and CEF.

Longitudinal associations between sleep measures at Observation 1 and changes in Receptive and Expressive Language, and in Cognitive EF to Observation 2 (Tables 2 and 4).

**TABLE 4:** Longitudinal effects.

Measures	Receptive vocabulary change rate	Expressive vocabulary change rate	CEF change rate
Model adjusted *R* ^2^	0.705**	0.259**	0.328**
Age Obs 1	7.01**	11.08**	0.00
SES	0.19	0.19	0.02
Sex	1.94	1.37	0.02
Measure at Obs 1	57.72**	31.11**	48.72**
Time asleep night	0.00	1.06	2.59
Sleep efficiency	0.33	0.36	3.05
Nap duration	3.71	14.71**	3.52
Number of naps	1.25	6.88*	0.18
Pandemic ECEC	6.30*	0.08	6.59*
Time asleep night × Age	0.06	0.91	1.06
Sleep efficiency × Age	1.47	0.73	0.00
Nap duration × Age	1.41	2.64	0.68
Number of naps × Age	0.39	1.53	0.01
*N*	164	164	181

The table lists *F* values and significance level; **p < 0.01, *p ≤ 0.05. Model adjusted *R* ^2^ is reported for the main effects model.

As seen in Table 4, Age at Observation 1 was a predictor of subsequent rate of change for vocabulary, with older children having a lower rate of change in Receptive Vocabulary but a higher rate of change in Expressive Vocabulary. Higher scores on all measures at Observation 1 associated with lower rates of change (likely reflecting a ceiling effect in the older children in the cohort). As reported previously (Davies et al., 2021), the number of days spend in daycare (Pandemic ECEC) positively associated with the growth in receptive vocabulary and in CEF. Sleep measures only associated with the rate of change in Expressive Vocabulary, with more frequent and shorter naps associated with a lower rate of change. This association was not moderated by age and age did not moderate the association between sleep parameters and Receptive Vocabulary or CEF.

## DISCUSSION

We investigated the association between two measures of cognitive development: vocabulary and cognitive EF, with various sleep parameters, in a cohort of 8–38‐month‐old children not attending daycare when the sleep measures were taken. We found that more frequent naps as well as shorter naps than expected for infants' age associate with smaller receptive vocabularies and lower scores in cognitive EF (when differences in age, SES and gender were accounted for), but not with expressive vocabulary. Night sleep had a modest association with receptive vocabulary and CEF, which is in line with evidence that a good proportion of night sleep variation is determined by variation in day sleep, in this age range (Nakagawa et al., 2016). Thus, we failed to replicate here the previously reported positive association between the frequency of naps and vocabulary, in a sample spanning the same age range as that of Horváth and Plunkett (2016). Moreover, we find an opposite pattern, where more frequent naps than expected by age, associate with smaller receptive vocabularies and we extend this finding to measures of EF. While the association between napping and cognition is not in line with Horváth and Plunkett (2016), our findings agree with experimental evidence showing that the number of times a child naps also reflects a cognitive need for sleep‐based information consolidation (Kurdziel et al., 2013; Kurth et al., 2016). Our findings therefore support a role for maturational factors in determining nap structure in childhood.

One proposed hypothesis suggested that the need for frequent napping during infancy reflected the protracted development of the hippocampus (Horváth & Plunkett, 2016; Mullally & Maguire, 2014). According to the two‐stages model of memory consolidation the hippocampus provides a short‐term locus for memory storage. That is, for information to be stored longer term and de‐contextualized, a “transfer” from hippocampal to cortical storage occurs (Marr, 1971; McClelland JL, McNaughton BL, O’Reilly RC. (1995)). There is now rich evidence that cortical consolidation of information happens during sleep (Buzsáki, 1998; Klinzing et al., 2019). The protracted development of the hippocampus may, therefore, limit the amount of information that can be temporarily stored, in children, which would then create the need for frequent sleep bouts (Gómez & Edgin, 2016; Nadel & Zola‐Morgan, 1984). As children grow older, and memory structures mature, day sleep consolidates in fewer naps of longer duration and eventually no naps. This may take longer for some children, which explains, in part, why they continue to nap later in childhood. While direct evidence for a link between hippocampus development and the need for frequent napping is currently unavailable, we provide here supportive indirect evidence needed to motivate further research.

**FIGURE 3:**
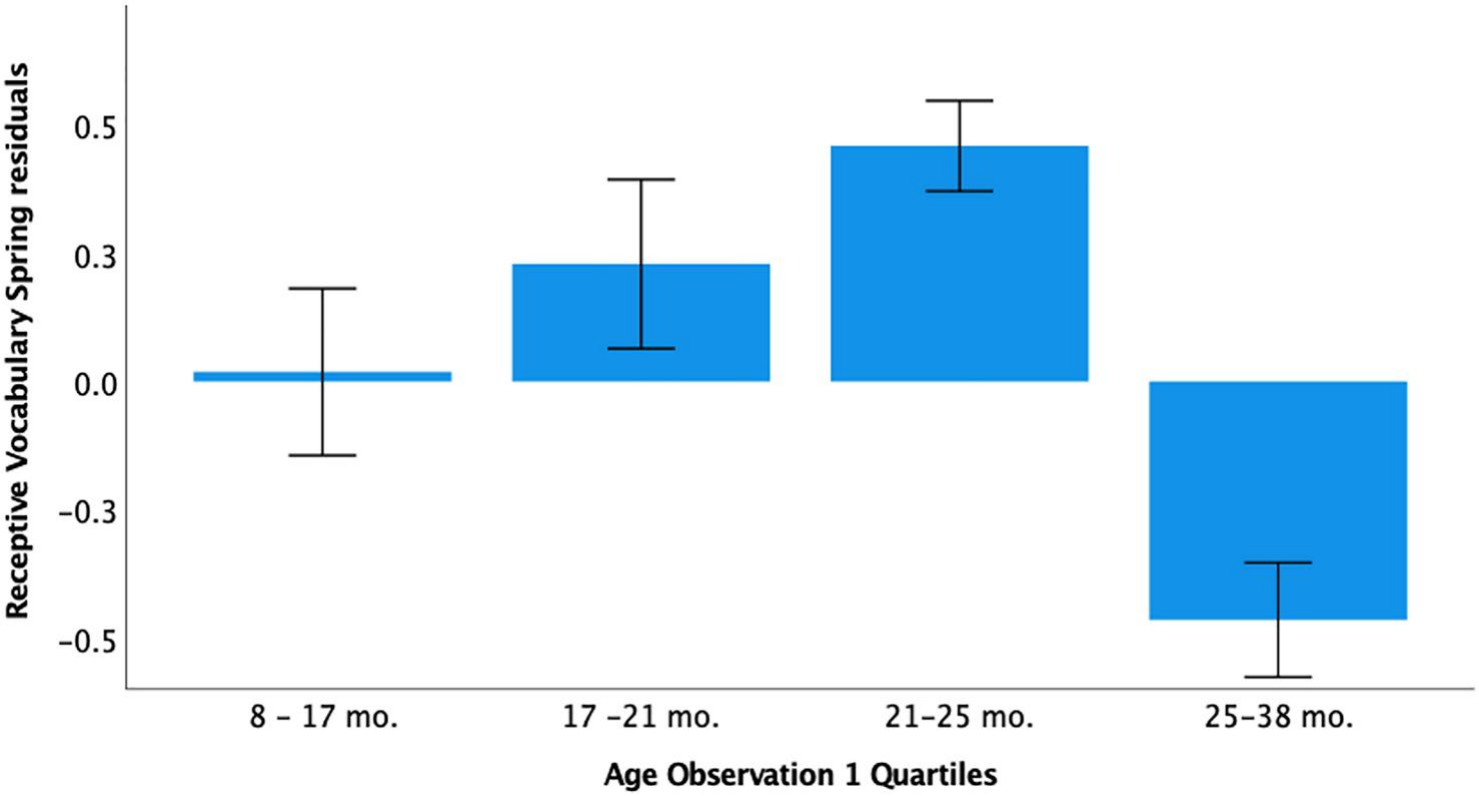
Napping once a day associates with lower vocabularies in both the younger and the older children in our cohort. *Y*‐axis depicts standardised residuals from the concurrent regression excluding the nap variable.

Age did not moderate the association between napping and EF, age did interact with nap variables to predict receptive vocabulary. As seen in Figure 4, this was due to older children showing a more prominent negative association between nap frequency and receptive vocabulary. There was no overall association between sleep measures and expressive vocabulary, but age moderated the association between nap frequency, nap length, sleep efficiency, and expressive vocabulary. In this case, a negative association between nap frequency and expressive vocabulary was only apparent in older children, with a positive association in the younger half of the group. It is therefore possible that the deficit and maturational model may contribute to these associations at different time points during development. Younger infants in our sample had a maximum of 3 naps, with only one infant having 3 naps and the remaining 2 naps a day. While this is considered normative for children older than 8 months of age (Staton et al., 2020), it may be that 2 naps remain insufficient for the youngest of infants. Despite the decrease in potential environmental interference with sleep patterns, in our cohort, the youngest of children were possibly not given the opportunity to fully match their day sleep needs. One way to illustrate the moderating effect of age is by looking at how standardised vocabulary changes with age in children that napped once (Figure 3). We chose children with 1 nap because this group had the widest age range (see figure S3), allowing us to compare vocabulary across 4 age quartiles. We can see that napping once, in the first quartile group, associates with smaller receptive vocabularies compared the 3rd quartile (e.g. *p* = 0.056, Dunnet correction) but the 3rd quartile had larger vocabularies than the 4th quartile (*p* < 0.001, Dunnet correction). It is interesting to note that although the age range of participants tested in Horváth and Plunkett (2016), was similar to ours (8–38‐months‐olds), the mean and median values were smaller (mean 18.3 months in Horvath and Plunkett compared to 20 months in our study, at Observation 1; median 16 months, compared to 19.6 months in our study). This may also have contributed to masking the effect of maturational factors in that particular study.

In line with our interpretation that nap structure reflects individual variation in cognitive ability, which is likely to be stable over time, we predicted that rate of change in vocabulary or EF would not associate with napping. This was the case for receptive vocabulary and for cognitive EF but not for expressive vocabulary. The change in the rate of expressive vocabulary growth over the 6 months that elapsed between Observation 1 and 2, was negatively associated with nap frequency and positively with nap duration. Why might expressive vocabulary behave differently than the other two measures? One possibility is that the protracted development in expressive language (see Fig S2), with many children non‐verbal at Observation 1, masks important underlying variation in the population, which becomes apparent at Observation 2.

We propose that we were able to reveal an association between sleep need and cognitive development because we carried out this study in a period of time when children were more likely to be allowed to fulfill their sleep needs. During the Covid‐19 lockdown, not only was daycare unavailable, but there was also a stark reduction in access to any group structured activities (e.g., playgroups, music groups, sports etc.). We suggest that, outside lockdown, these externally imposed routines curtail infants' day sleep, which means children sleep less than they need to, with negative consequences on learning. With fewer external perturbations, maturational factors made their presence felt, in our study. Although we have no direct evidence that the social distancing measures imposed in the UK interfered with children's day sleep, supporting evidence is provided by the fact that 26% of parents in our study reported changes in daytime sleeping routines from before to after lockdown. Importantly, it was those children that attended ECEC before lockdown for which parents reported more changes (decreases or increases) in daytime sleep, which supports our assumption that ECEC closure may have allowed children to return to a sleep pattern that reflected their cognitive needs.

Our findings provide an important perspective in the study of the links between sleep and cognition during development. In particular, we highlight the importance of taking into account the context in which sleep is measured, such as accounting for environmental factors that may interfere with a child's need for sleep. Variation in the influence of these factors might explain discrepancies between studies in whether they find or not an association between sleep structure and cognitive development (e.g. Mindell et al., 2017; Smithson et al., 2018). Moreover, we show that the age at which measures are taken influences the direction of association found. We therefore suggest, in line with previous work, that children's day sleep reflects both their cognitive needs and environmental factors that may interfere with these needs. One practical implications of our findings in that, rather than suggesting that children need to nap as frequently as possible (as Horváth & Plunkett, 2016 would imply), they should nap as frequently as they need to. Rather than using current normative data to organize children's sleep schedule, parents and early years educators could take a child‐led approach to napping, offering naps whenever the child signals a need to sleep.

Our study also adds to the growing literature reporting on the impact of lockdowns on child sleep. While many studies documented worsening of sleep quality in adults, associated with poorer mental health (Cellini et al., 2020; Zreik et al., 2021), there is less consensus in the developmental literature. In Zreik et al. (2021) in line with our findings, most parents reported no changes in 6‐ to 72‐months‐old's sleep and some reported overall improvement (also see Gupta et al., 2022). Moore et al. (2020) described longer sleep in children 5–11‐years‐old. Yet another study documented an overall shortening of total sleep time and an increase in night awakenings in 0–35‐month‐old children (Markovic et al., 2021). To explain discrepancies, it is critical to identify country or cohort‐specific parameters that may affect sleep. Differences in how lockdowns were implemented may manifest both in terms of availability of out‐of‐home activities as well as in more general measures that may affect family dynamics and mental health. In our study as well, we find a marginal effect of SES on day sleep, with those parents reporting a decrease in day sleep duration following the onset of lockdown having lower SES. Indeed, one shortcoming of our own study is that we have not collected data on parental working status during lockdown—whether parents had to work from home or not could have affected their ability to satisfy children's nap needs. A recent study found that working from home during the pandemic was associated with better sleep in both parents and children (Aishworiya et al., 2021). Finally, we acknowledge that the Covid‐19 pandemic was associated with tremendous mental and physical health challenges for families. While here we focus on lockdown‐related changes in access to childcare, screen time and access to outdoors activities, there were potentially many other factors we did not account for, and that may have contributed to both sleep patterns and cognitive development.

## CONCLUSION

We characterize here negative associations between the frequency of children's naps and concurrent receptive vocabulary and cognitive EF. This suggests that children with more advanced cognitive abilities require fewer naps, supporting the view that the frequency of naps changes during development in response to cognitive needs.

## AUTHOR CONTRIBUTIONS

**Teodora Gliga**: Conceptualization; data curation; formal analysis; funding acquisition; investigation; methodology; project administration; writing—original draft; writing—review and editing. **Alexandra Hendry**: Conceptualization; funding acquisition; investigation; methodology; writing—original draft; writing—review and editing. **Shannon P. Kong**: Data curation; formal analysis; investigation; methodology; writing—review and editing. **Ben Ewing**: Data curation; formal analysis; methodology; writing—review and editing. **Catherine Davies**: Conceptualization; funding acquisition; investigation; project administration; supervision; writing—review and editing. **Michelle McGillion**: Conceptualization; funding acquisition; investigation; project administration; writing—review and editing. **Nayeli Gonzalez‐Gomez**: Conceptualization; data curation; funding acquisition; investigation; methodology; project administration; supervision; writing—review and editing.

## CONFLICT OF INTEREST STATEMENT

The authors have declared that they have no competing or potential conflicts of interest.

## ETHICAL CONSIDERATIONS

This study received ethics approval from the Oxford Brooks University Research Ethics Committee. All procedures performed in this manuscript were in accordance with the 1964 Helsinki Declaration and its later amendments or comparable ethical standards. Participating caregivers provided informed consent at each time point, on behalf of themselves and their child.

## Supporting information

Supporting Information S1

## Data Availability

The data that support the findings of this study are openly available at https://osf.io/qbkwn.
