# Supplementary material for: More frequent naps are associated with lower cognitive development in a cohort of 8–38‐month‐old children, during the Covid‐19 pandemic
Source: JCPP Adv. 2023 Jul 27;3(4):e12190. doi: 10.1002/jcv2.12190 (PMC10694540; doi:10.1002/jcv2.12190)
Supplement: Supplementary file 1 — Supporting Information S1 [file JCV2-3-e12190-s001.docx]

**Supporting Information**

**Table S1**. Association between environmental and sleep variables, at Observation 1. The table indicates Pearson R values, none of which reached significance.

| Controlling for: age, SES, sex | Number of naps (0, 1, 2+) | Individual nap length | Night sleep efficiency | Time asleep at night |
| --- | --- | --- | --- | --- |
| Screen Use | -.066 | .001 | .002 | .028 |
| Outdoors Activities | -.067 | .003 | -.010 | -.014 |

**Appendix S1. Quantitative estimates of qualitative sleep data.**

This analysis investigates whether qualitative reports of changes in day and night sleep duration, following lockdown corresponded to **sleep parameters of interest, reported in Spring 2020.**

**Number of naps.** A multinomial logistic regression with Number of naps (0, 1, 2+) as outcome, Qualitative reports of day time sleep length as predictor and age, sex and SES as covariates yielded a significant model (chi-square = 370.58, p < .001). Nap evaluation was significantly associated with the number of naps (chi-square = 27.94, p < .001), with children whose parents reported longer day time sleep having more naps (see Table S2).

**Individual nap length.** An ANCOVAs, with nap length as outcome, Qualitative reports of changes in daytime sleep length (No change, Longer, Shorter) as predictor and age, sex and SES as covariates. Showed that reports of changes in nap length mapped on reported differences in nap duration (F(2, 475) = 14.79, p < .001), with children whose parents reported longer day time sleep having shorter naps (see Table S2).

**Time asleep at night.** An ANCOVAs, with nap time asleep at night as outcome, Qualitative reports of night time sleep length (No change, Longer, Shorter) as predictor and age, sex and SES as covariates yielded a significant association between qualitative reports and time asleep at night (F(2, 483) = 30.35, p <.001), with children whose parents reported longer night time sleep spending more time asleep at night (see Table S2)

**Sleep efficiency.** An ANCOVAs, with sleep efficiency as outcome, Qualitative reports of day time sleep length (No change, Longer, Shorter) as predictor and age and SES as covariates yielded a significant association between qualitative reports and sleep efficiency (F(2, 481) = 18.32, p <.001), with children whose parents reported longer night time sleep having higher sleep efficiency (see Table S2)

**Table S2.** Estimated marginal means from the models described above.

|  | **No change** | **Shorter sleep** | **Longer sleep** |
| --- | --- | --- | --- |
| **Nap length (h)** | 1.28 | 1.33 | 1.20 |
| **Number of naps** | 1.14 | .96 | 1.25 |
| **Time asleep night (h)** | 10.90 | 9.76 | 11.33 |
| **Sleep efficiency** | .97 | .93 | .99 |

**Appendix S2. Detailed description of the 4 indices contributing to the SES measure**

Neighbourhood deprivation index: Postcode data was used to compute an Index of Multiple Deprivation decile group using either the English indices of deprivation (Noble et al., 2019), the Northen Ireland Multiple Deprivation Measures (Power & Green, 2019), the Welsh Index of Multiple Deprivation (Welsh Government, 2019) or the Scottish Index of Multiple Deprivation (Scottish Government, 2020) as appropriate.

Income: Parents were asked to report their total household income from one of the following categories: 1: £0-£20k; 2: £21k-£30k; 3: £31k-£40k; 4: £41k-£50k; 5: £51k- £60k; 6: £61k-£70k; 7: £71k or over.

Parental education: Parents were asked to report their highest level of education completed from one of the following categories: 1: Primary school; 2: Secondary school (this is the minimum legal requirement for formal education in the UK), 3: Sixth form or college: 4: Vocational college; 5: Undergraduate: 6: Postgraduate: 7: MBA; 8: Doctoral degree. For single/widowed parents, only their scores were used in the analyses; otherwise, mean scores were computed based on both parents.

Parents’ occupational prestige: Parents were asked to report their occupation. This was converted into scores based on Hollingshead (1975) ranging from 1 to 9; whereby 1 is for cleaners or farm labourers, 5 is for clerical and sales workers, 7 is for owners of small businesses, managers, or journalists, and 9 is for executives, scientists, engineers, or large business owners. For single/widowed parents, only their scores were used in the analyses; otherwise, mean scores were computed based on all parents. If one parent was a full-time homemaker, the occupation score was based on the other working parent.


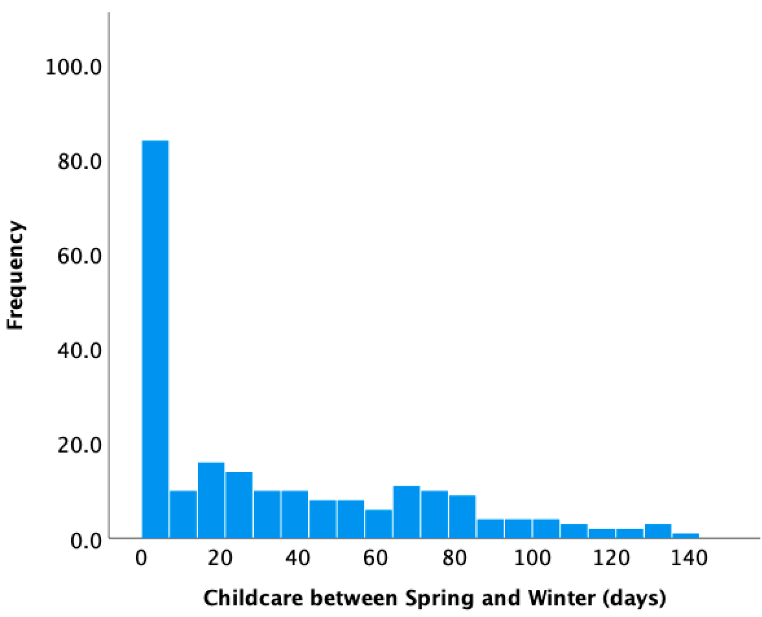


Figure S1 Distribution of days spent in childcare between Observation 1 and Observation 2


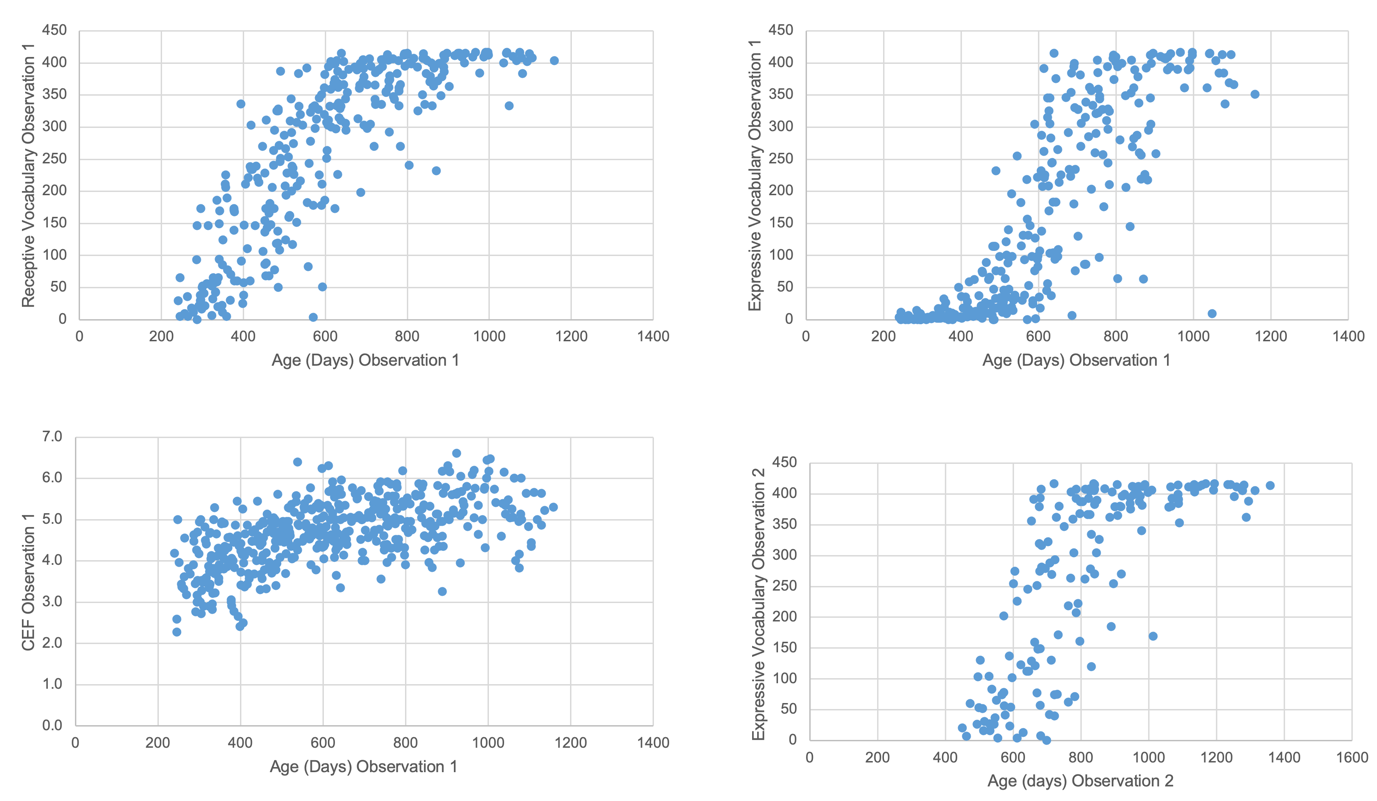


Figure S2. Developmental changes in main cognitive measures collected at Observation 1 & For Expressive Vocabulary at Observation 2.


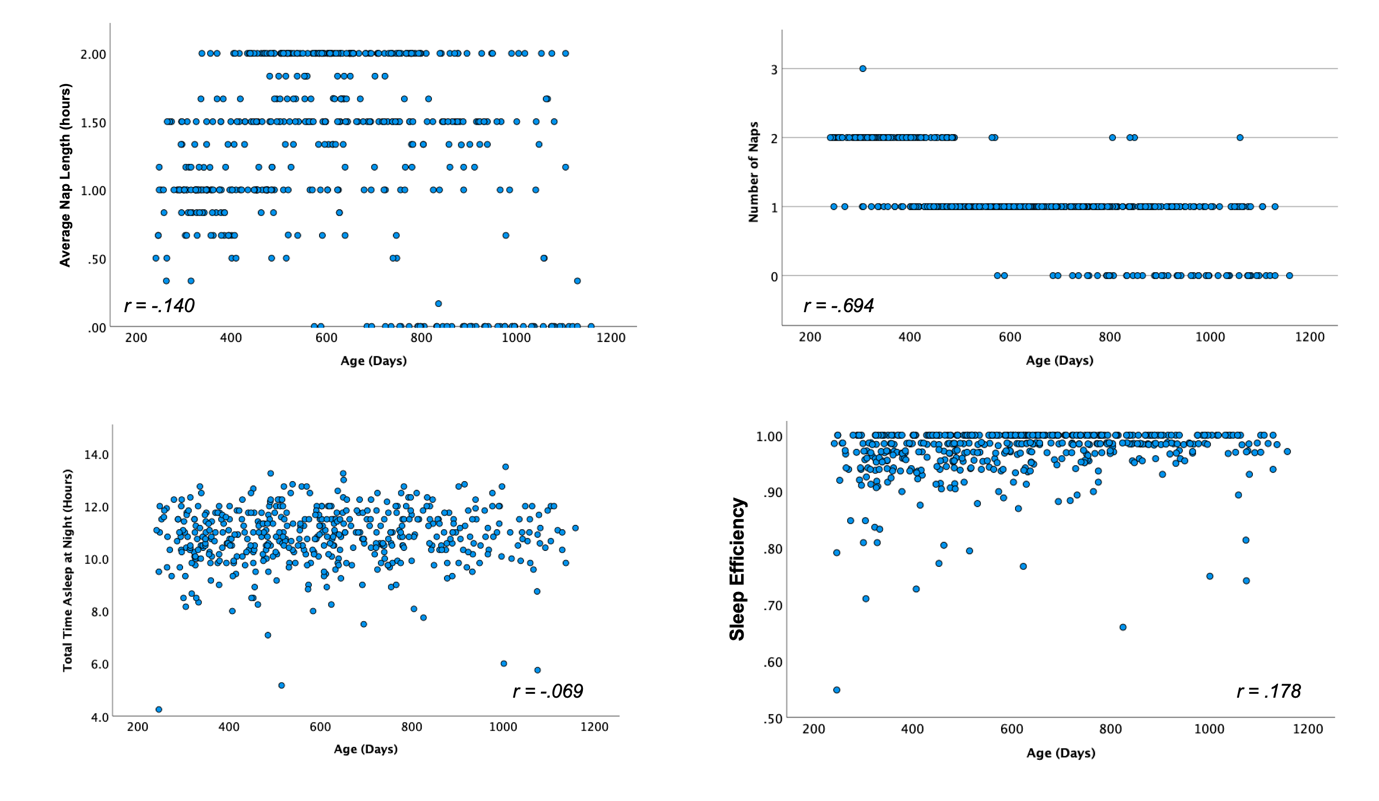


Figure S3. Developmental changes in main sleep measures collected at Observation 1.


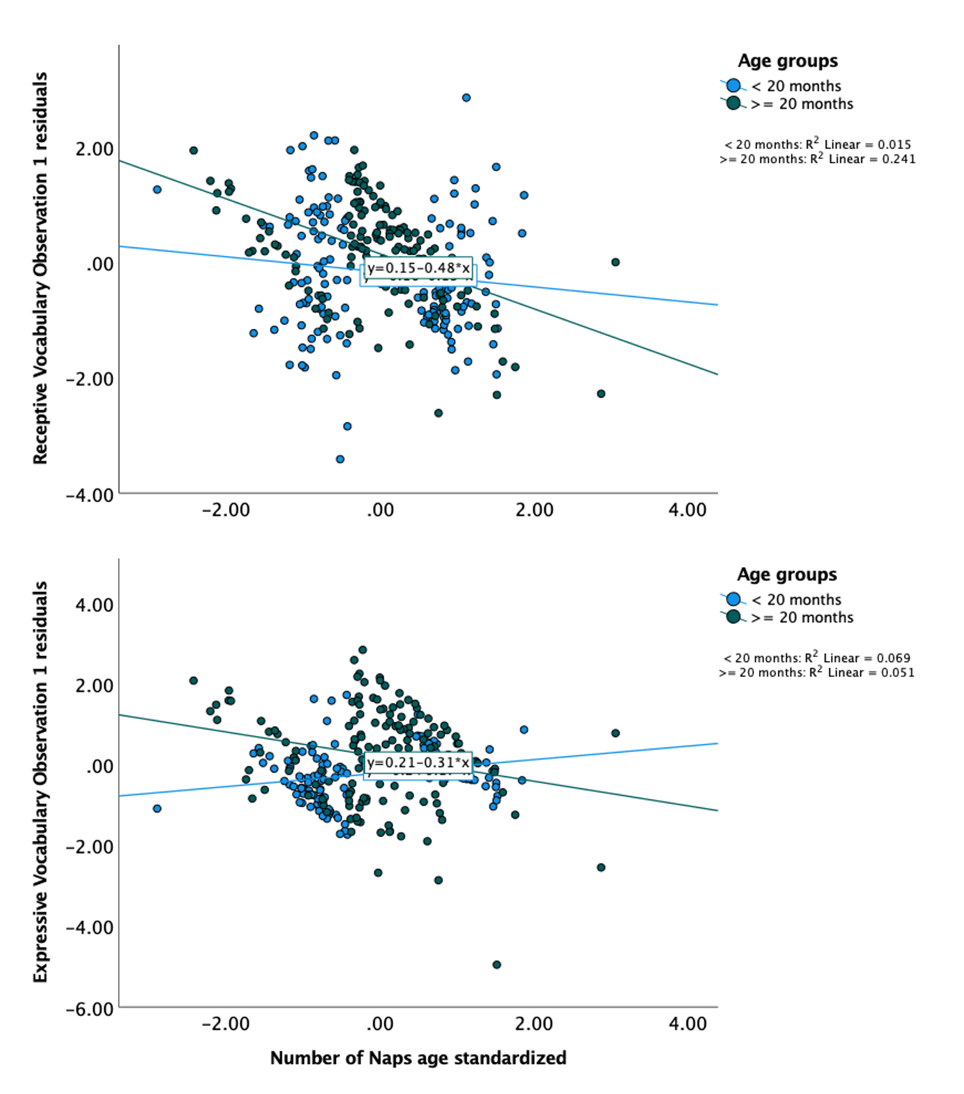


Figure S4. The association between the number of naps and Receptive vocabulary at Observation 1 as well as the change in Expressive vocabulary is moderated by age.
